# 非小细胞肺癌脑转移治疗进展

**DOI:** 10.3779/j.issn.1009-3419.2014.12.10

**Published:** 2014-12-20

**Authors:** 根 林, 海鹏 徐, 诚 黄

**Affiliations:** 350014 福州，福建省肿瘤医院胸部肿瘤内科，福建医科大学教学医院 Department of Medical Thoracic Oncology, Fujian Provincial Cancer Hospital, Fujian Medical University Teaching Hospital, Fuzhou 350014, China

**Keywords:** 肺肿瘤, 脑转移, 诊断, 治疗, Lung neoplsms, Brain metastasis, Diagnosis, Treatment

## Abstract

脑是非小细胞肺癌常见的转移部位，手术和放疗是以往脑转移治疗的基石，但近年来随着对肿瘤发生发展机制的认识深化，靶向治疗在脑转移治疗中开始崭露头角。本文主要针对一些相关热点问题如脑转移治疗手段等（手术、放疗、化疗、靶向治疗）进行简要述评。

## Abstract (en)

Metastatic tumors involving the brain are an important complication in the overall management of non-small cell lung cancers. Surgery and radiation remain the cornerstones of the therapy, however, the burgeoning knowledge of tumor biology has facilitated the entry of systemically administered therapies into the clinic. This review mainly summarizes the current applications of these data to surgery, radiation therapy, chemotherapy and targeted therapy.

肺癌最常见的远处转移部位之一是脑部，随着肺癌发病率的逐年上升，各种诊疗技术的提高，肺癌脑转移的发病率也随之增加。目前关于肺癌脑转移的诊疗成为研究热点之一，本文针对一些热点问题进行简要述评。

## 1 概述

### 1.1 流行病学

近年来脑转移发病率逐年攀升，这一现象的发生可能与磁共振成像（magnetic resonance imaging, MRI）影像学诊断技术广泛应用于临床以及肿瘤患者生存期延长有关^[1]^。一项瑞典研究纳入了15, 517例住院患者，从1987年至2006年，脑转移发生率从十万分之七上升到十万分之十四^[2]^。在原发肿瘤的构成中，肺癌脑转移最为常见，约占40%左右，其次是乳腺癌、结直肠癌、肾癌、恶性黑色素瘤等等^[1]^。非小细胞肺癌（non-small cell lung cancer, NSCLC）患者在首诊时约7.4%-10%患者存在脑转移，治疗过程中约30%-50%的患者会发生脑转移^[2-6]^，一项对975例早期接受手术诊疗的NSCLC患者的单中心回顾性分析^[6]^显示，患者脑转移5年累积发生率约为10%。

### 1.2 预后因素

以往脑转移患者的预后较差，总体的中位总生存时间（median overall survival, mOS）为2个月-3个月、1年生存率约为10%-20%^[1, 2]^。在解读脑转移相关临床试验的过程中，预后因素是一个重要的考量因素。目前已经发展了多个预后分级评分指数，主要包括了递归分割分析（recursive partition analysis, RPA）、放射外科评分指数（score index for radiosurgery, SIR）、Rotterdam评分、脑转移基础评分（basic score for brain metastases, BSBM）、Rades评分系统、预后等级评估（graded prognostic assessment, GPA）、Nomogram法等，其中以GPA应用最为广泛^[7, 8]^。2008年GPA分级系统的建立是基于一项大样本多中心回顾性研究，该研究分析3, 940例新诊断为脑转移患者，建立了以脑转移个数、卡氏评分、年龄、颅外转移有无等四个参数的数学预测模型，在GPA评分0-1、1.5-2.0、2.5-3.0和3.5-4.0等分级中，患者的mOS分别为3.0个月、5.5个月、9.4个月和14.8个月，显示出较好的临床应用价值，具体分级系统详见表 1^[8]^。另外，在以RTOG 9508临床试验数据分析中，GPA相对于RPA分级系统在预后良好的亚组患者中似乎显示出更好的预测效能^[9]^。但目前，不断有数据对脑转移个数作为独立预后因素提出质疑，认为肿瘤负荷（肿瘤总体积）可能更为重要^[10-12]^。

**1:** 预后分级评估 Graded prognostic assessment

Clinical feature	Score		
	0	0.5	1.0
No. of CNS metastases	> 3	2-3	1
KPS	< 70	70-80	90-100
Age	> 60	50-59	< 50
Extracranial metastases	Present	-	None
KPS: Karnofsky performance status; CNS: central nervous system.			

## 2 脑转移传统治疗手段

### 2.1 手术和放疗仍是目前脑转移癌的治疗基石

在美国国立综合癌症网络（National Comprehensive Cancer Network, NCCN）指南中，将脑转移个数作为治疗决策的重要考量因素之一，在1个-3个脑转移患者中，选择手术±全脑放疗（whole brain radiotherapy, WBRT）或立体定向放疗±WBRT往往根据临床实际情况而定，病灶可R0切除、肿瘤直径大于30 mm具有占位效应、位置表浅、放疗不敏感肿瘤患者可以首选手术治疗。在多发脑转移（≥4个）患者中，30 Gy/10 f全脑放疗是目前的标准治疗。

近年来，全脑放疗可能造成患者神经认知功能下降的远期不良反应越来越受到重视，而立体定向放射治疗（stereotactic radiosurgery, SRS）具有定位精确、剂量集中和安全快速等特点，并且具有非侵袭性、损伤相对较小和恢复快等优点，以往一些回顾性研究^[13-16]^提示SRS可以作为多发脑转移的备选方案，但目前仍缺乏随机对照的临床试验数据。2014年，日本学者Yamamoto等^[17]^公布了一项SRS治疗多发脑转移的大样本多中心前瞻性观察性临床研究结果，该临床试验主要入组标准包括：经增强MRI诊断为脑转移患者、脑转移病灶不超过10个、单病灶直径 < 3 cm且体积 < 10 mL、总体积 < 15 mL、无脑膜转移、Karnofsky体力状况评分（Karnofsky performance status, KPS）≥70分等，共入组1, 194例，均接受SRS治疗。在入组患者中，肺癌患者占了76%（*n*=912）。研究者按脑转移个数分为1个、2个-4个和5个-10个转移组，研究结果显示：三组mOS分别为13.9个月、10.8个月和10.8个月，单个脑转移生存时间要优于其他两组（*P*=0.000, 4），但2个-4个转移组与5个-10个转移组生存时间并无差别，另外，在肺癌这一亚组中，研究结果与总体结果基本一致，三组mOS分别为13.4个月、11.4个月和12.5个月。这是迄今为止第一个大样本观察SRS治疗多个脑转移患者的前瞻性临床研究，基于这个临床试验结果，一项Ⅲ期随机对照临床试验NCT01731704旨在入组多发脑转移患者（脑转移个数≥5个），评估SRS与WBRT的疗效及安全性。这一临床试验结果可能有助于进一步回答：在选择SRS或WBRT治疗脑转移时，脑转移个数是否是一个主要的治疗决策考量因素。

### 2.2 化疗

相对于放疗而言，以往认为血脑屏障以及脑组织高表达P-糖蛋白（P-glycoprotein, P-gp）可能是影响化疗作用的主要原因，在评估化疗的临床试验中往往将脑转移患者作为排除对象，相应地，以往评估化疗治疗脑转移的疗效的前瞻性临床试验数据很有限。但实际上当脑转移灶生长到一定程度时，脑转移灶的血脑屏障并不完整，MRI或电子计算机X线断层摄影（computed tomography, CT）增强扫描脑转移灶往往强化也说明了这一现象。

在不同的原发肿瘤中，脑转移患者的化疗疗效差别明显。在化疗敏感的肿瘤中，如生殖细胞肿瘤、绒毛膜癌、小细胞肺癌等，化疗可以起到根治的效果^[18-20]^，在化疗中度敏感肿瘤如乳腺癌，化疗在脑转移的近期疗效可达到50%左右^[21]^，但在化疗不敏感的肿瘤中，如NSCLC、恶性黑色素瘤，根据以往报道^[22]^，化疗治疗脑转移的总有效率（overall response rate, ORR）仅为15%左右。近年来，培美曲塞治疗NSCLC脑转移也逐渐引起重视，小样本Ⅱ期临床试验提示培美曲塞联合顺铂治疗无症状脑转移的NSCLC患者，颅内病灶、外周病灶以及总体的ORR分别为41.9%、34.9%和34.9%，mOS、中位无进展生存时间（median progression-free survival, mPFS）分别为7.4个月和4.0个月^[23]^，一项回顾性研究^[24]^结果提示培美曲塞与其他化疗药物相比，mOS要优于其他化疗药物（13.0个月*vs* 7.0个月，*P*=0.006）。

另一方面，即便是血脑屏障通透性良好的化疗药物在NSCLC脑转移治疗疗效也并不理想。血脑屏障通透性良好的化疗药物替莫唑胺在一项Ⅲ期临床试验（RTOG 0320）遭遇了失败，该研究是一项评估全脑放疗+SRS *vs*全脑放疗+SRS+替莫唑胺*vs*全脑放疗+SRS+厄洛替尼治疗NSCLC伴有1个-3个脑转移的Ⅲ期临床试验，由于入组困难，研究提前结束，三组mOS分别为13.4个月、6.3个月和6.1个月，中位至神经系统进展时间分别为8.1个月、4.6个月和4.8个月，但从上述有限的数据来看，在放疗基础上联合替莫唑胺并未改善生存，甚至有可能有害^[25]^。目前，第三代亚硝基脲药物福莫司汀治疗NSCLC脑转移的Ⅰ期/Ⅱ期临床研究也显示出一定疗效，单药有效率约为10%-24%，联合铂类ORR约为28%-37%^[26]^。

上述这些数据在一定程度上说明：当脑转移灶生长到一定程度时，化疗疗效可能更多取决于肿瘤的生物学特性而不是血脑屏障。因此，对于化疗敏感的NSCLC患者，化疗在NSCLC脑转移患者的姑息治疗中占有一定的地位。

## 3 靶向治疗在脑转移中的应用

### 3.1 表皮生长因子受体酪氨酸激酶抑制剂（epidermal growth factor receptor-tyrosine kinase inhibitors, EGFR-TKIs）

一项大样本回顾性研究表明*EGFR*突变患者可能好发脑转移，在314例初诊患者中，*EGFR*突变患者脑转移发生率高于*EGFR*野生型，同样，*EGFR*突变患者术后脑转移复发风险也高于*EGFR*野生型患者^[27]^。多项基础研究及临床研究^[28-33]^显示EGFR-TKIs（吉非替尼、厄洛替尼）脑脊液浓度远低于血浆浓度，但一项研究^[34]^应用同位素C^11^标记的厄洛替尼作为正电子发射断层成像（positron emission tomography, PET）的示踪剂获知厄洛替尼能在NSCLC颅内转移灶中的浓聚，类似的基础研究^[35, 36]^表明吉非替尼可选择性富集于肿瘤组织。可能正是上述这一特性，多项Ⅱ期/Ⅲ期临床试验结果显示常规剂量的EGFR-TKIs±全脑放疗治疗无症状脑转移显示出一定的疗效，详见表 2^[25, 37-45]^，这些临床试验设计绝大多数是单臂Ⅱ期临床试验，入组患者的选择从早期的无选择性到“EGFR-TKIs优势人群”再到专门入组*EGFR*活性突变患者，反映了EGFR-TKIs疗效预测分子标志物的认识历程，其中有数个临床试验值得进一步深入解读：两项临床试验入组*EGFR*活性突变的无症状脑转移患者，给予单药吉非替尼或厄洛替尼治疗，研究结果显示EGFR-TKIs对中枢神经系统转移灶的疗效并不差于外周病灶，ORR高达80%以上，mPFS分别为6.6个月和14.5个月^[38, 43]^。另一项临床试验^[45]^入组48例均为腺癌或*EGFR*活性突变患者，其中23例患者EGFR状态明确，在*EGFR*活性突变与*EGFR*野生型患者中，ORR 75% *vs* 33.3%、mPFS 15.2个月*vs* 4.4个月。这些数据提示我们：EGFR-TKIs治疗*EGFR*活性突变患者脑转移的疗效并不低于外周病灶，EGFR-TKIs无论是一线治疗、二线治疗或者与放疗联合，可能也需要选择*EGFR*突变患者。

**2:** EGFR-TKIs治疗肺癌脑转移的临床研究 Clinical studies of EGFR-TKIs for brain metastases in lung cancer

Intervention	Published time	Design	*EGFR* mutation	Number of patients	ORR/DCR（%）	mPFS/OS（mo）
Gefitinib	2004^[37]^	Prospective single arm phase Ⅱ	Unselected	41	10/27	3/5
	2007^[40]^	Prospective single arm phase Ⅱ	Adenocarcinoma Asian	40	38/83	9/15
	2009^[42]^	Prospective single arm phase Ⅱ WBRT+Gefitinib	Adenocarcinoma	21	81/95	10.0/13.0
	2013^[38]^	Prospective single arm phase Ⅱ	*EGFR* mutation	41	87.8/97.6	14.5/21.9
Erlotinib	2011^[39]^	Retrospective Erlotinib±XRT	Unselected	17 *EGFR* mutation 39 *EGFR* wild-type	82/100 0/78	11.7/12.9 3.1/5.8
	2013^[45]^	Prospective single arm phase Ⅱ	Adenocarcinoma or *EGFR* mutation	48 8 *EGFR* mutation 15 *EGFR* wild-type	58.4/75.1 75/87.5 33.3/46.7	9.7/18.9 15.2/37.5 4.4/18.4
	2013^[44]^	Prospective single arm phase Ⅱ WBRT+Erlotinib	Unselected	40	83/86	8/11.8
	2013^[25]^	Prospective randomized phase Ⅲ WBRT+SRS *vs* WBRT+SRS+Erlotinib *vs* WBRT+SRS+temozomide	Unselected	126	NR	NR 8.1/13.4 4.8/6.1 4.6/6.3
Gefitinib or erlotinib	2009^[41]^	Prospective single arm phase Ⅱ	Never smoking Adenocarcinoma Asian	23	70/74	7.1/18.8
	2012^[43]^	Prospective single arm phase Ⅱ	*EGFR* mutation	28	83/94	6.6/15.9
EGFR-TKI: epidermal growth factor receptor-tyrosine kinase inhibitor; ORR: overall response rate; DCR: disease control rate; mPFS: median progress-free survival; OS: overall survival; WBRT: whole-brain radiation therapy; XRT: external-beam radiation therapy; SRS: stereotactic radiosurgery; NR: not reported.						

目前单药EGFR-TKIs治疗NSCLC脑转移的临床数据主要局限于无症状脑转移患者，今后临床试验研究方向是进一步积累有症状脑转移患者的治疗疗效及安全性数据，探索EGFR-TKIs与全脑放疗头对头比较以及与其他模式联合应用的最佳治疗模式等等。目前，有数项Ⅱ期/Ⅲ期临床试验正在试图进一步回答上述问题，详见表 3。

**3:** EGFR-TKIs治疗肺癌脑转移的在研试验 Ongoing trials of EGFR-TKIs therapy in NSCLC patients with brain metastases

ClinicalTrials.gov identifier	Design	Intervention	*EGFR* mutation
NCT01887795	Prospective randomized phase Ⅲ	WBRT *vs* WBRT+erlotinib	Unselected
NCT01724801	Prospective randomized phase Ⅲ	WBRT *vs* icotinib	*EGFR* mutation
NCT01578668	Prospective single arm phase Ⅱ	Erlotinib+chemotherapy (pemetrexed plus carboplatin)	Adenocarcinoma
NCT01218529	Prospective single arm phase Ⅱ	WBRT+lapatinib	Unselected
NSCLC: non-small cell lung cancer.			

以往脑转移一线治疗进展后有效治疗手段较为匮乏，而在*EGFR*突变脑转移患者接受EGFR-TKIs治疗进展后，脉冲式EGFR-TKIs给药模式可能是另一个有效治疗手段。一项回顾性研究^[46]^纳入9例*EGFR*突变脑转移患者，既往均接受过EGFR-TKIs治疗后中枢神经系统病变进展（新发或进展），其中4例患者进行耐药后脑脊液检测EGFR T790M，结果均为阴性，给予厄洛替尼1, 500 mg每周一次治疗，9例患者6例再次获得部分缓解（partial response, PR），PFS 1.8个月-14.5个月，mPFS 2.7个月，副作用可控、可耐受。但这种治疗模式仍需进一步积累数据进行充分评估，更为关键的是，我们对*EGFR*突变脑转移的耐药机制还知之甚少，一些有限数据显示EGFR突变脑转移患者EGFR-TKIs继发性耐药的T790M比例明显低于外周病灶耐药模式，这提示中枢神经系统耐药模式可能有别于外周模式。另外，由于耐药病灶再次活检常有困难（尤其是颅内病灶），使得这个问题更加突出，这是限制脑转移患者EGFR-TKIs继发性耐药后治疗选择的一个瓶颈。

### 3.2 其他靶向药物

#### 3.2.1 ALK抑制剂

以往一系列克唑替尼临床试验也入组脑转移患者，但研究结果显示克唑替尼在脑转移病灶的ORR并不高，与外周病灶的高缓解率有较大差别。另外，与EGFR-TKIs相类似，克唑替尼的脑脊液浓度也低于血浆浓度，两者比约为0.26%^[47]^。但在二代ALK抑制剂Ceritinib治疗ALK阳性伴脑转移的晚期NSCLC的Ⅰ期/Ⅱ期临床试验中，其中入组基线伴有脑转移患者124例，在这124例患者中，既往接受过ALK抑制剂治疗患者有10例可评价疗效，Ceritinib治疗有效4例，另外既往未接受过ALK抑制剂治疗患者有4例可评价疗效，Ceritinib治疗有效3例。上述研究虽然样本量较小，但提示Ceritinib在既往ALK抑制剂治疗失败的患者还是未接受过ALK抑制剂治疗的患者中初步表现出抗肿瘤活性，有较高的脑转移灶缓解率^[48]^。

#### 3.2.2 贝伐珠单抗

在早期大多数贝伐珠单抗临床试验中，由于颅内出血的风险，脑转移的患者均被排除在外。最近的一些数据^[49]^显示脑转移患者接受贝伐珠单抗治疗的安全性良好，一项回顾性研究汇总了13项随机对照临床试验中入组的131例经处理的中枢神经系统转移患者，这些患者均持续接受贝伐珠单抗治疗，只有1例患者（0.8%）出现2级脑出血。另一项PASSPORT临床试验^[50]^入组了115例脑转移患者，也未发现2级或更高中枢神经系统出血的不良事件。在疗效上，一些个案报道或小样本前瞻性临床试验数据表明贝伐珠单抗对脑转移治疗有潜在临床应用价值，但仍需进一步积累数据^[51]^。

#### 3.2.3 免疫靶向

伊匹单抗是CTLA-4单克隆抗体，在早期一些有限的数据^[52, 53]^提示伊匹单抗对黑色素瘤脑转移患者有一定疗效。2010年美国临床肿瘤学会（American Society of Clinical Oncology, ASCO）报道了一项评估伊匹单抗治疗黑色素瘤脑转移患者疗效的前瞻性研究结果，51例不需要类固醇处理的脑转移患者接受伊匹单抗治疗，总体疗效：4例PR，5例病情平稳，疾病控制率18%；对颅内病灶进行单独评估，5例PR、6例SD^[54]^。因此，这些有限的数据提示伊匹单抗对黑色素瘤中枢神经系统病灶与外周病灶可能具有相似的抗肿瘤活性，但目前在NSCLC患者中，尚缺乏相关临床数据。同样，PD-1单抗于PD-L1单抗在NSCLC脑转移患者中是否具有治疗活性仍有待进一步研究。

## 4 临床试验规范

近年来，随着靶向治疗在NSCLC中取得突破性进展，系统性治疗手段治疗脑转移的临床试验也开始逐渐增多，但相关临床试验的质量参差不齐。针对这一现状，神经系统肿瘤疗效评价协助组（Response Assessment in Neuro-Oncology group, RANO）对脑转移临床试验提出规范意见，主要包括以下几个方面的内容：①脑转移患者不应常规作为临床试验入组人群的排除标准；②在入组人群选择方面，建议最好入组单一肿瘤类型；如果临床试验入组多种肿瘤患者，研究药物的治疗靶点应该尽可能相同，并作为分层因素，另外，应保证不同肿瘤病例数足够充分进行统计学分析；③在临床试验主要研究终点中，近期疗效评价强烈推荐增强MRI为标准影像学评价，MRI检测参数应标准化；推荐以MRI的3D容积测量作为疗效评价标准方法，但这一建议需进一步积累数据；建立鉴别放射性脑坏死及肿瘤进展的有效影像学技术；④在以PFS为临床试验主要研究终点中，建议同时采用颅内PFS（intracranial PFS, iPFS）、总PFS（overall PFS, oPFS），应在临床试验方案实施中明确定义两者，iPFS可用于手术、放疗等局部治疗手段的评价，oPFS应常规应用于系统治疗手段；另外，在研究终点中还涉及到神经认知功能、生活质量评估等；⑤针对评价系统治疗手段的临床试验，建议患者仅有颅内病灶进展，在方案设计中可继续系统治疗手段±局部治疗，但PFS以iPFS记录为准^[55, 56]^。

## 5 总结

脑转移发病率有逐年增高趋势，肺癌患者易发生脑转移^[57]^，治疗疗效仍面临巨大挑战。手术、全脑放疗、SRS在脑转移传统治疗中仍占有重要位置，多个临床试验证实EGFR-TKIs在*EGFR*突变脑转移患者的治疗疗效与外周病灶相当，但在有症状脑转移患者以及与其他治疗手段的最佳联合模式仍有待于临床试验数据积累。脑转移的治疗已经进入手术、放疗以及靶向药物等多种治疗手段并存的个体化治疗新时代。随着脑转移发病机制的深入研究，一些新的治疗靶点得以关注，但临床试验质量参差不齐，仍有待进一步规范，今后不应再将脑转移患者作为临床试验的常规排除标准。

## References

[1] Tabouret E, Chinot O, Metellus P (2012). Recent trends in epidemiology of brain metastases: an overview. Anticancer Res.

[2] Smedby KE, Brandt L, Backlund ML (2009). Brain metastases admissions in Sweden between 1987 and 2006. Br J Cancer.

[3] Sorensen JB, Hansen HH, Hansen M (1988). Brain metastases in adenocarcinoma of the lung: frequency, risk groups, and prognosis. J Clin Oncol.

[4] Komaki R, Cox JD, Stark R (1983). Frequency of brain metastasis in adenocarcinoma and large cell carcinoma of the lung: correlation with survival. Int J Radiat Oncol Biol Phys.

[5] Schouten LJ, Rutten J, Huveneers HA (2002). Incidence of brain metastases in a cohort of patients with carcinoma of the breast, colon, kidney, and lung and melanoma. Cancer.

[6] Hubbs JL, Boyd JA, Hollis D (2010). Factors associated with the development of brain metastases: analysis of 975 patients with early stage non-small cell lung cancer. Cancer.

[7] Gaspar L, Scott C, Rotman M (1997). Recursive partitioning analysis (RPA) of prognostic factors in three Radiation Therapy Oncology Group (RTOG) brain metastases trials. Int J Radiat Oncol Biol Phys.

[8] Sperduto PW, Berkey B, Gaspar LE (2008). A new prognostic index and comparison to three other indices for patients with brain metastases: an analysis of 1, 960 patients in the RTOG database. Int J Radiat Oncol Biol Phys.

[9] Sperduto PW, Shanley R, Luo X (2014). Secondary analysis of RTOG 9508, a phase 3 randomized trial of whole-brain radiation therapy versus WBRT plus stereotactic radiosurgery in patients with 1-3 brain metastases; poststratified by the graded prognostic assessment (GPA). Int J Radiat Oncol Biol Phys.

[10] Likhacheva A, Pinnix CC, Parikh NR (2013). Predictors of survival in contemporary practice after initial radiosurgery for brain metastases. Int J Radiat Oncol Biol Phys.

[11] Bhatnagar AK, Flickinger JC, Kondziolka D (2006). Stereotactic radiosurgery for four or more intracranial metastases. Int J Radiat Oncol Biol Phys.

[12] Baschnagel AM, Meyer KD, Chen PY (2013). Tumor volume as a predictor of survival and local control in patients with brain etastases treated with Gamma Knife surgery. J Neurosurg.

[13] Chang WS, Kim HY, Chang JW (2010). Analysis of radiosurgical results in patients with brain metastases according to he number of brain lesions: is stereotactic radiosurgery effective for multiple brain metastases?. J Neurosurg.

[14] Ojerholm E, Lee JY, Kolker J (2014). Gamma Knife radiosurgery to four or more brain metastases in patients without prior intracranial radiation or surgery. Cancer Med.

[15] Kim HS, Koh EJ, Choi HY (2012). Multiple gamma knife radiosurgery for multiple metachronous brain metastases associated with lung cancer: survival time. J Korean Neurosurg Soc.

[16] Jairam V, Chiang VL, Yu JB (2013). Role of stereotactic radiosurgery in patients with more than four brain metastases. CNS Oncol.

[17] Yamamoto M, Serizawa T, Shuto T (2014). Stereotactic radiosurgery for patients with multiple brain metastases (JLGK0901): a multi-institutional prospective observational study. Lancet Oncol.

[18] Rustin GJ, Newlands ES, Begent RH (1989). Weekly alternating etoposide, methotrexate, and actinomycin/vincristine and cyclophosphamide chemotherapy for the treatment of CNS metastases of choriocarcinoma. J Clin Oncol.

[19] Kristensen CA, Kristjansen PE, Hansen HH (1992). Systemic chemotherapy of brain metastases from small-cell lung cancer: a review. J Clin Oncol.

[20] Logothetis CJ, Samuels ML, Trindade A (1982). The management of brain metastases in germ cell tumors. Cancer.

[21] Rosner D, Nemoto T, Lane WW (1986). Chemotherapy induces regression of brain metastases in breast carcinoma. Cancer.

[b22] 22Handbook of Clinical Neurology, Vol. 121 (3rd series)

[23] Barlesi F, Gervais R, Lena H (2011). Pemetrexed and cisplatin as first-line chemotherapy for advanced non-small-cell lung cancer (NSCLC) with asymptomatic inoperable brain metastases: a multicenter phase Ⅱ trial (GFPC 07-01). Ann Oncol.

[24] Fan Y, Huang Z, Fang L (2013). Chemotherapy and EGFR tyrosine kinase inhibitors for treatment of brain metastases from non-small-cell lung cancer: survival analysis in 210 patients. Oncol Targets Ther.

[25] Sperduto PW, Wang M, Robins HI (2013). A phase 3 trial of whole brain radiation therapy and stereotactic radiosurgery alone versus WBRT and SRS with temozolomide or erlotinib for non-small cell lung ancer and 1 to 3 brain metastases: Radiation Therapy Oncology Group 0320. Int J Radiat Oncol Biol Phys.

[26] Addeo R, Zappavigna S, Luce A (2013). Chemotherapy in the management of brain metastases: the emerging role of fotemustine for patients with melanoma and NSCLC. Expert Opin Drug Saf.

[27] Shin DY, Na II, Kim CH (2014). *EGFR* mutation and brain metastasis in pulmonary adenocarcinomas. J Thorac Oncol.

[28] Togashi Y, Masago K, Fukudo M (2011). Efficacy of increased-dose erlotinib for central nervous system metastases in non-small cell lung cancer patients with epidermal growth factor receptor mutation. Cancer Chemother Pharmacol.

[29] Masuda T, Hattori N, Hamada A (2011). Erlotinib efficacy and cerebrospinal fluid concentration in patients with lung adenocarcinoma developing leptomeningeal metastases during gefitinib therapy. Cancer Chemother Pharmacol.

[30] Broniscer A, Panetta JC, O'Shaughnessy M (2007). Plasma and cerebrospinal fluid pharmacokinetics of erlotinib and its active metabolite OSI-420. Clin Cancer Res.

[31] Jackman DM, Holmes AJ, Lindeman N (2006). Response and resistance in a non-small-cell lung cancer patient with an epidermal growth factor receptor mutation and leptomeningeal metastases treated with high-dose gefitinib. J Clin Oncol.

[32] Togashi Y, Masago K, Fukudo M (2010). Cerebrospinal fluid concentration of erlotinib and its active metabolite OSI-420 in patients with central nervous system metastases of non-small cell lung cancer. J Thorac Oncol.

[33] Fukuhara T, Saijo Y, Sakakibara T (2008). Successful treatment of carcinomatous meningitis with gefitinib in a patient with lung adenocarcinoma harboring a mutated EGF receptor gene. Tohoku J Exp Med.

[34] Weber B, Winterdahl M, Memon A (2011). Erlotinib accumulation in brain metastases from non-small cell lung cancer: visualization by positron emission tomography in a patient harboring a mutation in the epidermal growth factor receptor. J Thorac Oncol.

[35] Chen Y, Wang M, Zhong W (2013). Pharmacokinetic and pharmacodynamic study of Gefitinib in a mouse model of non-small-cell lung carcinoma with brain metastasis. Lung Cancer.

[36] Mckillop D, Partridge EA, Kemp JV (2005). Tumor penetration of gefitinib (Iressa), an epidermal growth factor receptor tyrosine kinase inhibitor. Mol Cancer Ther.

[37] Ceresoli GL, Cappuzzo F, Gregorc V (2004). Gefitinib in patients with brain metastases from non-small-cell lung cancer: a prospective trial. Ann Oncol.

[38] Wu YL, Zhou C, Cheng Y (2013). Erlotinib as second-line treatment in patients with advanced non-small-cell lung cancer and asymptomatic brain metastases: a phase Ⅱ study (CTONG-0803). Ann Oncol.

[39] Porta R, Sanchez-Torres JM, Paz-Ares L (2011). Brain metastases from lung cancer responding to erlotinib: the importance of *EGFR* mutation. Eur Respir J.

[40] Wu C, Li YL, Wang ZM (2007). Gefitinib as palliative therapy for lung adenocarcinoma metastatic to the brain. Lung Cancer.

[41] Kim JE, Lee DH, Choi Y (2009). Epidermal growth factor receptor tyrosine kinase inhibitors as a first-line therapy for never-smokers with adenocarcinoma of the lung having asymptomatic synchronous brain metastasis. Lung Cancer.

[42] Ma S, Xu Y, Deng Q (2009). Treatment of brain metastasis from non-small cell lung cancer with whole brain radiotherapy and Gefitinib in a Chinese population. Lung Cancer.

[43] Park SJ, Kim HT, Lee DH (2012). Efficacy of epidermal growth factor receptor tyrosine kinase inhibitors for brain metastasis in non-small cell lung cancer patients harboring either exon 19 or 21 mutation. Lung Cancer.

[44] Iuchi T, Shingyoji M, Sakaida T (2013). Phase Ⅱ trial of gefitinib alone without radiation therapy for Japanese patients with brain metastases from *EGFR*-mutant lung adenocarcinoma. Lung Cancer.

[45] Welsh JW, Komaki R, Amini A (2013). Phase Ⅱ trial of erlotinib plus concurrent whole-brain radiation therapy for patients with brain metastases from non-small-cell lung cancer. J Clin Oncol.

[46] Grommes C, Oxnard GR, Kris MG (2011). "Pulsatile" high-dose weekly erlotinib for CNS metastases from *EGFR* mutant non-small cell lung cancer. Neuro Oncol.

[47] Costa DB, Kobayashi S, Pandya SS (2011). CSF concentration of the anaplastic lymphoma kinase inhibitor crizotinib. J Clin Oncol.

[48] Kim DW, Mehra R, Tan DS (2014). Ceritinib in advanced anaplastic lymphoma kinase (ALK)-rearranged (ALK+) non-small cell lung cancer (NSCLC): Results of the ASCEND-1 trial. J Clin Oncol.

[49] Besse B, Lasserre SF, Compton P (2010). Bevacizumab safety in patients with central nervous system metastases. Clin Cancer Res.

[50] Socinski MA, Langer CJ, Huang JE (2009). Safety of bevacizumab in patients with non-small-cell lung cancer and brain metastases. J Clin Oncol.

[51] De Braganca KC, Janjigian YY, Azzoli CG (2010). Efficacy and safety of bevacizumab in active brain metastases from non-small cell lung cancer. J Neurooncol.

[52] Margolin KA, Di Giacomo AM, Maio M (2010). Brain metastasis in melanoma: clinical activity of CTLA-4 antibody therapy. Semin Oncol.

[53] Weber JS, Amin A, Minor D (2011). Safety and clinical activity of ipilimumab in melanoma patients with brain metastases: retrospective analysis of data from a phase 2 trial. Melanoma Res.

[54] Lawrence D, Hamid O, McDermott D (2010). Phase Ⅱ trial of ipilimumab monotherapy in melanoma patients with brain metastases. J Clin Oncol.

[55] Lin NU, Lee EQ, Aoyama H (2013). Challenges relating to solid tumour brain metastases in clinical trials, part 1: patient population, response, and progression. Lancet Oncol.

[56] Lin NU, Wefel JS, Lee EQ (2013). Challenges relating to solid tumour brain metastases in clinical trials, part 2: neurocognitive, neurological, and quality-of-life outcomes. A report from the RANO group. Lancet Oncol.

[57] Jiang R, Ma CH, Zhu ZL (2014). Application of detecting cerebrospinal fluid circulating tumor cells in the diagnosis of meningeal metastasis of non-small cell lung cancer. Zhongguo Xian Dai Shen Jing Ji Bing Za Zhi.

